# Maternal kidney adaptation and function in pregnancy: review and perspectives

**DOI:** 10.1152/function.044.2026

**Published:** 2026-10-01

**Authors:** Jessica F. Hebert, Kevin Burfeind, Michael P. Hutchens

**Affiliations:** ^1^Division of Nephrology and Hypertension, Department of Medicine, 6684 Oregon Health & Science University, Portland, Oregon, United States; ^2^Department of Anesthesiology and Perioperative Medicine, Oregon Health & Science University, Portland, Oregon, United States; ^3^Operative Care Division, Portland Veterans Administration Medical Center, Portland, Oregon, United States

**Keywords:** interorgan communication, kidney, placenta, pregnancy, women’s health

## Abstract

Pregnancy imposes considerable physiological demands on the maternal kidney, requiring coordinated structural, hemodynamic, and molecular adaptations to maintain homeostasis and support fetal development. This review synthesizes renal changes throughout normal and abnormal gestation, emphasizing the dynamic interactions among maternal, placental, and fetal systems. These adaptations are driven by integrated hormonal pathways, including the renin-angiotensin system and vasodilatory mediators, as well as emerging mechanotransductive processes that enable the kidney to sense and respond to increased flow and pressure. Bidirectional signaling between the placenta and maternal kidney aligns maternal physiology with fetal demand. Dysregulation of these processes leads to complications such as preeclampsia, gestational hypertension, and pregnancy-associated acute kidney injury, with lasting consequences for maternal and offspring health. Key gaps remain in defining kidney and placental interactions, renal immune and mechanosensory roles, and accurate biomarkers of renal function in pregnancy. Advancing research in these areas is critical to improving diagnosis and outcomes in renal and pregnancy-related disorders.

## Graphical abstract

**Figure:**
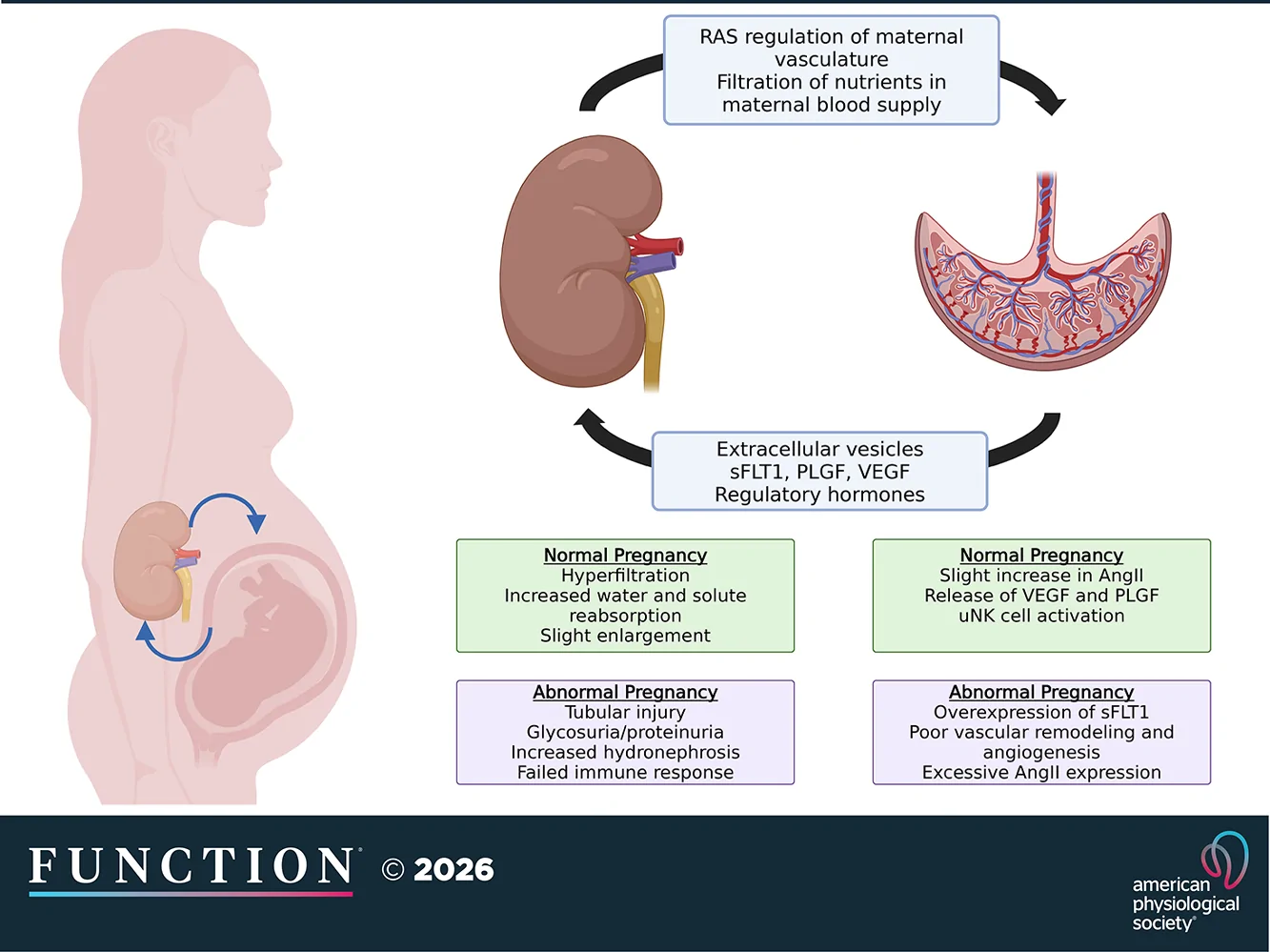

## INTRODUCTION

The maternal kidney undergoes profound changes during pregnancy in response to the increased physiological demands imposed by gestation. These adaptations are critical in maintaining homeostasis for both the mother and the developing fetus. Disruptions in this delicate balance can contribute to adverse maternal and offspring outcomes, both immediate and long-term, underscoring the importance of this field.

The dynamic interactions between maternal and fetal systems and the long-term impact of pregnancy on subsequent maternal and offspring health are critical to understanding the pathology of pregnancy complications, identifying potential treatments to improve pregnancy outcomes, and exploring the developmental origins of health and disease. In this review, we will address the interactions of the maternal kidney and the fetus through several lenses. First, we will discuss the normal function of the maternal kidney during gestation and the renal adaptations necessary to sustain the demands of placental and fetal development. Here, adaptations in the maternal kidney are viewed as responses to maternal physiological changes, as increased blood volume leads to a higher glomerular filtration rate (GFR) and altered electrolyte handling. We will also address some ways pre-pregnancy renal disease may affect subsequent pregnancy, as well as the response of the fetus and the role of maternal-fetal interactions. This perspective allows us to consider renal adaptations as driven by fetal requirements, possibly mediated by placental signals. Bidirectional communication between the placenta and maternal kidney coordinates resource allocation for optimal fetal development. Finally, we will discuss some unresolved questions regarding the interaction between the maternal kidney and the fetoplacental unit.

The connection between the kidney and pregnancy has long been alluded to in historical sources. Swelling and inflammation often observed in pregnancy were collectively termed “edema” by Hippocrates in the 5th century BC (1). In the 1st century AD, Soranus of Ephesus opined that the uterus was like the kidney in that both helped evacuate certain wastes from the body (2). In 1840, Pierre Rayer described proteinuria in eclampsia, which was further defined by John Lever in 1943, who associated increased urinary protein levels with maternal seizures during pregnancy (3, 4). This renal malfunction and application of kidney disease specifically to pregnancy as a transient disease gradually disappearing postpartum is what we now know as preeclampsia (5). Preeclampsia has become one of the preeminent diseases studied during gestation as it affects up to 8% of pregnancies in the United States (6); recurrence of preeclampsia in subsequent pregnancies is reported to be 20%–50% (7–9). However, maternal comorbidities add complexity to renal function during gestation. These diseases include maternal hypertension, chronic kidney disease, cardiovascular disease, diabetes, or immune system diseases. Renal damage occurring pre-pregnancy, whether acute or sustained, impacts gestational outcomes by increasing the risk for preterm birth, growth restriction, and other complications (10–12). Poor fertility and miscarriage are far more common in people with pre-pregnancy disease that impacts renal function, as are preterm birth and low birthweight (13). Gestational diabetes may also impair maternal renal function. Arising specifically during pregnancy, gestational diabetes is still associated with pre-pregnancy risk factors, including higher pre-pregnancy BMI, obesity, advanced maternal age, or previous instances of gestational diabetes. A history of gestational diabetes increases the risk of future maternal type 2 diabetes and chronic kidney disease (14). Gestational diabetes is not associated with changes in GFR during pregnancy, however (15). A recent systematic review well illustrates the broad use of numerous experimental animal models demonstrating that maladaptive renal responses contribute directly to pregnancy complications rather than simply reflecting downstream consequences of disease (16). Animal models of preeclampsia, including mice, rats, sheep, and nonhuman primates, have provided important mechanistic insights into altered renal hemodynamics, impaired glomerular and tubular function, angiogenic imbalance, immune activation, angiotensin II type 1 receptor autoantibodies, oxidative stress, and dysregulation of the intrarenal renin-angiotensin system as contributors to hypertension and renal injury during pregnancy. Each model uniquely addresses varying stages of pregnancy at which complications may uniquely disrupt the normal progression of gestation, whether through endothelial dysfunction, placental malperfusion, inflammation, or angiogenic imbalance (17–21). Additional animal models of altered maternal kidney function resulting in poor pregnancy outcomes, including fetal growth restriction and longitudinal maternal renal dysfunction, have also been subject to investigation and may improve our understanding of the complex interaction of maternal and fetal demands in pregnancy (22–24). These studies have clarified how disruption of normal gestational renal adaptations contributes to preeclampsia and other pregnancy complications and suggest novel therapeutic targets.

Vascular function throughout the body is affected by renal function, particularly in pregnancy, when the kidneys must adapt to increased blood volume and the release of factors associated with blood vessel tone and development. One of the biggest drivers of physiological changes in pregnancy affecting the kidney is the placenta, a pregnancy-specific exchange organ that connects the maternal and fetal circulations (25). Inadequate placentation leads to fetal underdevelopment and maternal endotheliosis, which may be affected by and also impact maternal renal dysfunction.

## BALANCING MATERNAL AND FETAL NEEDS IN PREGNANCY

Although the maternal body externally supports fetal development, it is the fetus, placenta, and maternal habitus working in concert during pregnancy that may determine the success or failure of gestation. Throughout gestation, the maternal kidney influences time-dependent changes in function: a marked increase in GFR and renal blood flow in early pregnancy, fine-tuning of filtration and reabsorptive processes in mid- to late pregnancy, and a gradual return to pre-pregnancy values postpartum. The kidney’s ability to adapt to the demands of pregnancy is remarkable and critical to reproductive success.

### Maternal Kidney Changes in Normal Pregnancy

The maternal body rapidly undergoes a series of physiological alterations in response to pregnancy as early as 6 wk gestation, with many of these changes requiring adaptation in renal function. One of the primary changes in early pregnancy is the increase in total body blood volume (26). The primary driver of blood volume expansion is the increase in plasma volume; whole blood is roughly 55% plasma by volume pre-pregnancy, and plasma is ∼90% water. Plasma volume expands due to increased renal reabsorption of sodium and water paired with reduced vascular resistance throughout the body (27). Plasma volume rises ∼30%–50% from pre-pregnancy to term, with the most rapid increase occurring in the second trimester; plasma volume remains relatively steady from 32 wk onward (28–30). Red blood cell (RBC) volume in the blood also increases due to plasma volume and elevated erythropoietin levels, which rise in response to placental production of progesterone (31). Fetal and placental demand for oxygen helps drive the surge in progesterone and RBCs; however, as red blood cell volume only increases ∼20%–30%, it is the increase in plasma volume that is primarily responsible for increased total blood volume (32). As such, the increase in the ratio of plasma to RBCs may result in an anemia-like state, with lower hemoglobin levels as a total percentage of blood, compounded by fetal iron demand (33). Iron supplementation, either oral supplements or administered intravenously, may be required to mitigate this imbalance and support fetal growth (34, 35). Postpartum, rapid diuresis occurs, resulting in a reduction of plasma volume to normal pre-pregnancy levels. The drivers of plasma volume expansion in pregnancy are not well understood, but key adaptations include increased epithelial sodium channel (ENaC) activity, decreased sodium-chloride cotransport (NCC) activity, and vasodilation as promoted by the renin-angiotensin system to improve placental perfusion and provide for fetal growth (25). This occurs despite elevated nitric oxide (NO) signaling and an increase in the vasodilator relaxin, which, while promoting vasodilation, also increases natriuresis, which is not observed in pregnancy (36, 37).

Although blood volume increases significantly during pregnancy, blood pressure in normal pregnancy usually remains below pre-pregnancy levels due to a decrease in peripheral and renal vascular resistance starting in the first trimester (38). Maternal blood pressure also modulates throughout gestation in response to changes in blood pressure-determining factors, such as systemic vascular resistance, fetoplacental nutrient requirements, and cardiac output. Vascular resistance declines as a response to increased systemic vasodilation caused by rising circulating levels of relaxin, progesterone, NO, and prostaglandins in the first trimester (39–42). This dilation of peripheral blood vessels is critical for placental development and, therefore, fetal development. Cardiac output increases in early pregnancy in response to rapidly expanding blood volume; however, vasodilation often lowers blood pressure until well into the second trimester. Elevated blood pressure may be observed in the third trimester due to increased cardiac output and peak plasma volume expansion; however, high blood pressure in pregnancy [defined as >140/90 mmHg by the American College of Obstetricians and Gynecologists (ACOG) (43)] in conjunction with proteinuria or elevated markers of organ dysfunction, particularly renal dysfunction, is generally diagnosed as preeclampsia (44, 45) (46). If diagnosed before 34 wk, high blood pressure and proteinuria are classified as early-onset preeclampsia (see Links between Maternal Renal Function and Complications of Pregnancy) and are associated with fetal growth restriction and placental malfunction, with a potential for severe maternal and fetal complications. Late-onset preeclampsia, occurring after 34 wk of gestation, usually results in a less severe phenotype with early or near-term delivery (47). Blood pressure generally normalizes to similar levels pre-pregnancy within 3 to 4 mo following delivery, and previous guidance indicated that the cure for preeclampsia was the delivery of the placenta (48). However, postpartum preeclampsia, characterized by high blood pressure in conjunction with proteinuria, heart failure, serum abnormalities, or neurological symptoms, is observed in 0.3%–27.5% of pregnancies and may occur up to 6 wk following birth, most commonly in the 48 h after (49). The variance in the percentage of affected pregnancies is attributed to a high incidence of going undetected. Postpartum preeclampsia may progress to include seizures and it is then defined as postpartum eclampsia; this was found to occur in nearly 15% of cases of postpartum preeclampsia in a clinical cohort (50). Elevated blood pressure during and following pregnancy can indicate serious issues on the horizon for the health of both mother and fetus.

Glomerular filtration rate (GFR) is a measure of the flow rate of fluid as filtered by the kidney, and is a useful tool to estimate renal function (51). An estimated glomerular filtration rate (eGFR) is calculated from serum creatinine measured on a standard metabolic panel blood test combined with considerations for age and sex, though other metabolites have been suggested as better serum markers for eGFR calculations, including cystatin C (52). Creatinine is a 113 Da protein produced as a standard waste product by the muscles. Higher blood creatinine levels indicate poor renal function, as creatinine is not excreted in the urine. As plasma volume and cardiac rate increase during pregnancy, the kidneys must adjust to increased blood flow and filtration demand. Therefore, eGFR increases during pregnancy by 20%–50% in humans and rodent models, peaking during the third trimester (53–57). eGFR decreases slightly near term, before gradually returning to pre-pregnancy levels by 2 mo following delivery, aided by the loss of extra blood volume postpartum (15, 58). Despite elevated GFR, however, natriuresis does not increase during pregnancy, exhibiting a similar effect as elevated NO (25).

Other minor but significant changes also occur in and to the maternal kidney during pregnancy. The steep rise in circulating estrogen released by the placenta causes maternal renal enlargement of ∼1–1.5 cm. Hydronephrosis is detectable in 90% of pregnancies by 28 wk’ gestation, though it is clinically asymptomatic in all but ∼3% of cases (59). To accommodate the increased blood volume and flow, the ureters and renal pelvis dilate. Progesterone, estrogen, and relaxin induce smooth muscle relaxation in the urinary tract, leading to these observed physiological changes in kidney expansion (60, 61). Of note, the right kidney generally enlarges more than the left. The asymmetrical expansion is common and normal; it is due to the physical compression from the expanding and dextrorotating uterus through gestation (62). Slight elevations in proteinuria and glycosuria may be present throughout normal pregnancy due to increased GFR. However, elevated levels of either may indicate gestational complications. Protein levels exceeding 300 mg/day may indicate renal dysfunction and, coupled with high blood pressure, may be diagnosed as preeclampsia (63). Blood glucose levels over 95 mg/dL fasting or 120 mg/dL 2 h after a meal may be observed in patients with gestational diabetes (64). Although slight increases in kidney size and excretion of protein and glucose are normal and generally resolve after delivery, more significant shifts may indicate problems and warrant early delivery or intervention. Normal adaptations of the maternal renal system in response to pregnancy are illustrated in Fig. 1.

**Figure 1.**
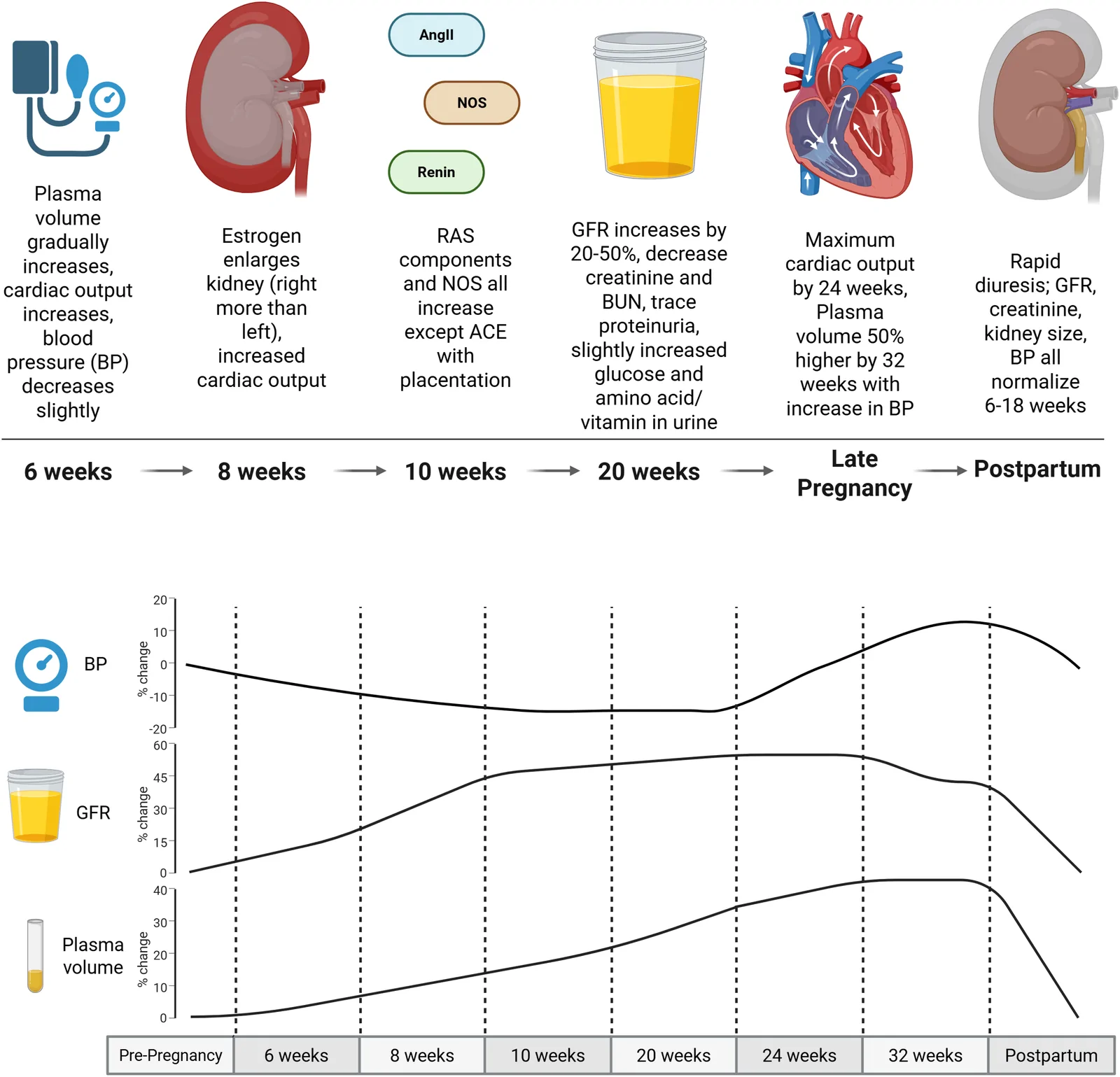
Overview of maternal renal-influenced changes over the course of gestation. Maternal kidney response to rapidly rising hormonal levels and fetoplacental demand primarily by increasing plasma volume and glomerular filtration rate and aiding with vasodilation, causing reduced blood pressure as early as the first trimester of pregnancy. Up until term, the maternal kidney continues to respond to the changing state of pregnancy and renormalizes to pre-pregnancy baseline conditions postpartum. Figure created with a licensed version of BioRender.com.

### Mechanisms of Maternal Renal Adaptation in Uncomplicated Pregnancy

Over the course of pregnancy, kidneys adapt to rapidly changing fetal and placental demands, requiring different parts of the nephron to adjust their function in response to hemodynamic and volumetric changes. As eGFR and plasma volume increase during pregnancy, creatinine, sodium, potassium, and other serum solutes decrease, resulting in critical changes in chemistry regulated by the maternal kidney. Urine output increases, and so does the concentration of urinary solutes as filtered by the kidney. Table 1 outlines the most critical changes from the nonpregnant to the pregnant state. Pregnancy distinctly alters each nephron segment: the glomerulus, the proximal tubule, the Loop of Henle, the distal tubule, and the collecting duct each undergoes gestational adaptation. The major effects of pregnancy on each nephron segment are illustrated in Fig. 2.

**Figure 2.**
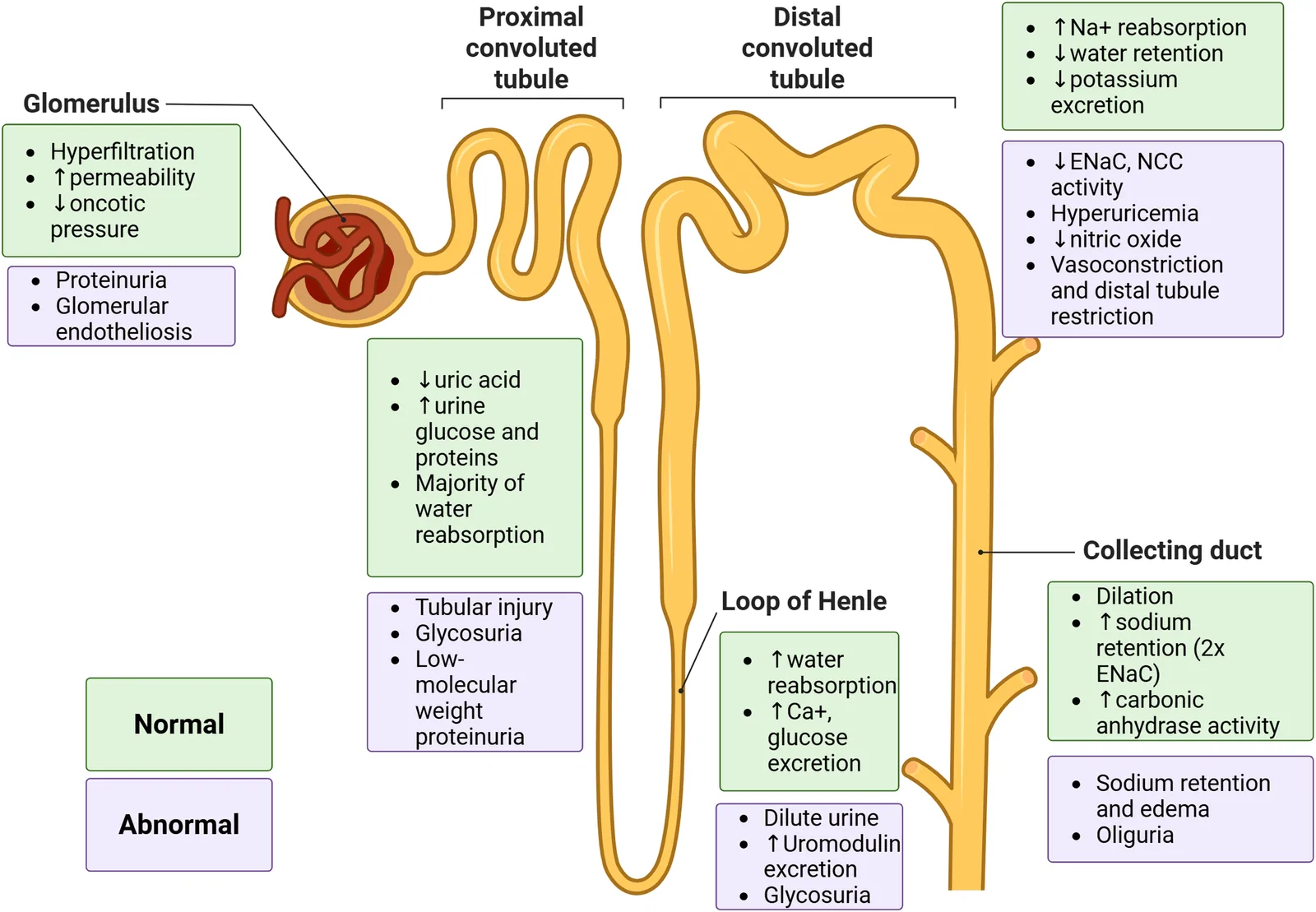
Comparison of nephron section level changes in normal pregnancy vs. abnormal pregnancy as compared with nonpregnant filtration. Figure created with a licensed version of BioRender.com.

**Table 1.** Normal pre-pregnancy and pregnancy values for standard renal parameters and blood and urine chemistries

Parameter	Nonpregnant Normal Value	Pregnant Normal Value
Renal		
Kidney length, cm	10–12 (65)	11–14 (53)
eGFR, mL/min/1.73m^2^	>90 (66)	120–150 (53)
BP, mmHg	≈120/80 (47)	Early: <120/80; Late: <140/90 (47)
Blood		
Plasma osmolality, mosmol/kgH_2_O	275–295 (67)	270–280 (68)
Serum sodium, mEq/L	135–145 (69)	132–142 (68)
Serum potassium, mEq/L	3.5–5.0 (69)	3.4–4.8 (68)
Serum bicarbonate, mEq/L	22–26 (70)	18–26 (68)
Serum creatinine, mg/dL	0.5–1.1 (71)	0.4–0.8 (68)
Serum albumin, mg/dL	3.8–5.0 (72)	2.3–5.1 (73)
Blood urea nitrogen, mg/dL	10–20 (71)	3–13 (68)
Uric acid, mg/dL	3.9–5.3 (74)	2–6 (68)
Urine		
Total protein, mg/day	<150 (75)	<300 (75)
Protein:creatinine, mg/mmol	<20 (76)	<30 (76)
Glucose, mmol/L	0–0.8 (77)	<2.7 (78)

The glomerular filtration barrier (glomerular endothelium, basement membrane, and podocytes around the glomerular capillaries) imposes cooperative charge and size restriction on the passage and filtration of solutes into the nephron (79). Two forces critical to glomerular filtration are altered by pregnancy. Increased glomerular capillary pressure due to elevated plasma volume and cardiac stroke volume increases renal blood flow and GFR and can also increase permeability, allowing larger or more proteins to pass through the glomerular barrier into the nephron (80–82).

Solutes passing through the glomerulus next reach the proximal convoluted tubule (PT). The PT reabsorbs glucose, amino acids, and most salts by both diffusion and active transport, including uric acid (83, 84). In pregnancy, the proximal tubule lengthens in response to increased fluid volume and renal flow (85, 86). With increased surface area, transporters for reabsorption including sodium glucose transporters (SGLTs), organic anion transporters (OATs), and sodium hydrogen exchangers (notably NHE3) are upregulated (86–88). The presence of high levels of glucose or protein in the urine indicates that the maximal reabsorptive capacity of the PT transporters has been exceeded. However, some glucose and protein may still pass into the urine during normal pregnancy, at levels higher than pre-pregnancy, due to increased blood volume and the passage of additional glucose and protein from the glomerulus, resulting in reduced renal tubular reabsorption amid increased filtration demand and hormonal influences. In addition to solute reabsorption, the proximal tubule allows most water to return to the bloodstream by osmosis. Extremes in pH disrupt homeostasis and must be tightly controlled, a challenge that is exacerbated by increased plasma volume and ion load. Carbonic anhydrase activity in the proximal tubule balances pH by aiding in bicarbonate reabsorption and ion transport (89).

The loop of Henle is the only segment to reside entirely within the renal medulla. The osmotic gradient formed on both sides of this U-shaped limb alternately absorbs water with salt impermeability (descending limb) and absorbs salts by active transport (ascending limb). This results in a concentrated fluid at the bottom of the loop. As the fluid ascends, salts are removed, contributing to the high osmolarity of the medulla and perpetuating the gradient-driven action of this part of the nephron. Calcium and glucose may also be excreted within the loop of Henle; however, the increase in net urinary glucose is more due to the overall increase in glucose than to a failure of reabsorption (78, 90). This is also true for calcium: compared with the nonpregnant state, the amount of calcium excreted into the urine would be considered hypercalciuria, but this is an adaptive function of the loop of Henle observed in uncomplicated pregnancy (91). Calcium excretion may be up to 2–3 times higher in pregnancy, which could potentially spell kidney stone formation in the nonpregnant state, but increased circulating magnesium and citrate help inhibit formation, so the risk is not significantly elevated in normal pregnancy (92).

Concentrated urine then travels to the distal convoluted tubule (DCT), where the majority of sodium and calcium reabsorption occurs. The DCT plays a key role in maintaining pH, electrolyte balance, and blood pressure. This is primarily achieved through aldosterone, which stimulates the activity of the sodium-chloride symporter (NCC) and the epithelial sodium channel (ENaC) via mineralocorticoid receptors to increase reabsorption of sodium and excretion of potassium (93), which can be further stimulated by angiotensin II (94). Angiotensin II, estrogen, and aldosterone activation as observed in pregnancy would suggest a very high retention of sodium, but nitric oxide, progesterone, and GFR increase function as antagonists to counteract some of this activity (95–98). Although ENaC expression is markedly elevated during pregnancy, progesterone-mediated modulation of mineralocorticoid receptor (MR) signaling limits distal potassium secretion, facilitating sodium retention while maintaining potassium homeostasis (99). Increased plasma volume raises renal plasma flow and therefore GFR, whereas circulating progesterone and nitric oxide levels are heightened due to placental release. In pregnancy, substantial remodeling of distal nephron transport takes place to support maternal plasma volume expansion while maintaining electrolyte homeostasis. Although the DCT is acted upon by several natriuretic and antinatriuretic factors during pregnancy that would seem in conflict, the net effect remains to maintain volume expansion and retain sodium despite a significant increase in renal filtration rate and net volume (85, 100).

The last stop for filtrate before reaching the ureters and bladder is the collecting duct (CD), where urine is further concentrated. Aquaporin channels respond to vasopressin to reabsorb water from the filtrate back into the blood; without vasopressin, the CD is largely water-impermeable, demonstrating the need for hormonal regulation (101). In pregnancy, increased levels of circulating relaxin and progesterone cause the collecting ducts and ureters to dilate to compensate for the higher volume of excretion. Rising progesterone levels throughout the third trimester also significantly upregulate CD potassium reabsorption (25). The collecting ducts also help retain sodium in the blood due to a two- to threefold gestational increase in CD ENaC (86, 102). Elevated carbonic anhydrase activity in the collecting ducts helps regulate urinary pH during pregnancy. With the significant increase in maternal fluid volume, this additional method of controlling pH in filtered urine is essential for maintaining blood pH stability.

One of the key mechanisms regulating renal function in pregnancy is the renin-angiotensin system (RAS). Almost all RAS factors are elevated throughout the body during pregnancy, except angiotensin-converting enzyme (ACE) (103, 104). In the classic renin-angiotensin system cascade, angiotensinogen released by the liver is cleaved by renin created by the renal juxtoglomerular cells to form angiotensin I, and then cleaved again by ACE to form angiotensin II (AngII), the functional product that induces vasoconstriction, increased water and salt retention, and increased sympathetic activity (105). Circulating RAS factors (namely AngII) and intrarenal RAS levels may both affect renal function (106). AngII is a potent vasoconstrictor that alters the glomerular filtration rate by decreasing blood flow through systemic vasoconstriction and by affecting the renal efferent arteriole (107). Local renal levels of AngII have been reported to be significantly higher than in circulation, even in pregnancy, with levels in the renal interstitium and tubules reported to be as much as 100–1,000 times higher than in plasma (108–110). Renal angiotensin 1 (AT1) receptors are highly responsive to this elevated RAS state in pregnancy. AngII acts on the DCT to promote sodium and water reabsorption, which is necessary to maintain increased plasma volume (111, 112). Renin rises in circulation during pregnancy, particularly in the first trimester, which is critical to help maintain stable blood pressure under the reduction of peripheral resistance with increasing blood volume in normal pregnancy (113). Maternal RAS is additionally stimulated by the rising levels of aldosterone and progesterone produced by the developing placenta, which helps regulate renal response to fetal and placental demand. Understanding intrarenal and circulating RAS dynamics during pregnancy is essential for defining the mechanisms that coordinate vascular regulation between the kidney and the systemic circulation.

Mechanotransduction plays a key role in the renal response to increased flow and pressure by taking the force applied and translating it to signaling pathways, particularly for channel adaptations (114). Recent research, including studies on Piezo1 and Piezo2, highlights how mechanosensing channels and cytoskeletal changes contribute to renal adaptation (115, 116). The sensitivity of release of arginine vasopressin has been linked to Piezo1 mechanosensors in the collecting duct (117). Arginine vasopressin levels generally fluctuate with the tonicity of bodily fluids. However, in pregnancy, arginine vasopressin release mechanisms seem to adjust to new volume levels throughout gestation, leading to higher fluid retention (118). Piezo2 appears to be more expressed in juxtaglomerular cells and is involved in regulating renin production and, therefore, may be associated with critical adaptations in RAS necessary for vascular regulation (116). Apical mechanosensors in the proximal tubule detect increased flow and pressure through their microvilli, activating integrin signaling to induce stress and thereby adjust for higher absorption (119). Renal mechanotransduction is likely part of the maternal renal adaptations during pregnancy and a promising area for exploration. Recently, it was found that physiological concentrations of pregnancy-related hormones, including estrogens, progesterone, cortisol, testosterone, and placental growth hormone, did not change the expression levels of organic anion transporters related to renal clearance in proximal tubule cell cultures across pregnancy trimesters; the authors suggest that mechanosensitive changes to flow may be more responsible (120). Piezo1 and Piezo2 have been examined as critical mechanosensors in the uterus during gestation: Piezo1 is critical to the stimulation of labor and contractions and uterine artery vasodilation in response to pressure in the smooth muscle, and Piezo2 is found in the sensory neurons of the cervix and vagina to assist in labor (121–124). Therefore, the discovery of an expanded role for Piezo1 and Piezo2 in the kidney, with the changing renal hemodynamics during pregnancy, is likely on the horizon. These pressure-sensing mechanisms offer a new perspective on how the kidney responds dynamically to the changing physiological demands of pregnancy.

### Links between Maternal Renal Function and Complications of Pregnancy

Abnormal renal function in pregnancy can be attributed to numerous etiologies, both from pregestational and gestational effects. Maternal hypertension in pregnancy may arise de novo during gestation or may already be present beforehand. Hypertension in pregnancy is not uncommon, affecting ∼16% of all pregnancies with a higher prevalence with advanced maternal age (defined as over 35 yr old) (125) and in Black and American Indian women (126). Although it may lead to increased risks of future maternal cardiovascular disease, the primary reason to monitor maternal hypertension is to ensure that it is not associated with onset of preeclampsia or eclampsia, organ damage, or stroke.

The elevation of maternal blood pressure on its own (generally more than one reading above 140/90) without evidence of maternal organ dysfunction is diagnosed as gestational hypertension (127). About 15%–25% of cases of gestational hypertension progress to preeclampsia, which is characterized by high maternal blood pressure in conjunction with proteinuria or markers of organ dysfunction. It may arise from several maternal or fetal determinants. Hypertension after the 20th week of pregnancy in conjunction with proteinuria, particularly high creatinine:protein ratio, often with excessive edema, has historically been the primary diagnostic criterion for preeclampsia (6, 128). Additional factors are increasingly considered as criteria, including impairment of the hepatic endothelium leading to HELLP (hemolysis, elevated liver enzymes and low platelet count) syndrome and serum levels of soluble fms-like tyrosine kinase 1 (sFlt1), placental growth factor (PLGF), pregnancy-associated plasma protein A (PAPP-A), soluble endoglin (sEng), or glycosylated fibronectin (129–131). The exact etiology of preeclampsia is not well understood; however, proposed mechanisms include abnormal placental development characterized by impaired spiral artery remodeling and placental ischemia, which promote the release of antiangiogenic and proinflammatory mediators into the maternal circulation as early as the first trimester (103, 132, 133). These changes lead to endothelial activation, vascular dysfunction, and subsequent impairment of multiple organ systems (134).

Dysregulation of the renin-angiotensin system (RAS), particularly in response to changing hormonal adaptations during pregnancy, remains an area of active investigation in understanding preeclampsia as it is key to placental vasculature development, uterine spiral artery remodeling and uteroplacental blood flow regulation, and maternal cardiovascular response (103, 135). RAS responds to fluctuations in blood pressure and volume, as well as renal perfusion; changes to all of these are common in pregnancy in response to plasma volume expansion and vasodilation. Renal AT1 receptors are highly responsive to this elevated RAS state in pregnancy, which may further contribute to an exaggerated vasoconstrictive response and endothelial dysfunction as observed in preeclampsia (136, 137). In pregnancy, AngII and aldosterone work together to promote sodium retention, plasma volume expansion, and uteroplacental perfusion. AngII stimulates the release of aldosterone, which both increases circulating renin, the key cleaving enzyme of angiotensinogen to form more AngII, and acts on the DCT to promote sodium and water reabsorption, which is necessary to maintain increased plasma volume (111, 112). In the nonpregnant state, elevated aldosterone and AngII would increase blood pressure and result in worse renal function; however, vascular sensitivity to AngII in normal pregnancy is reduced so blood pressure remains low (138, 139). However, circulating aldosterone levels above the 75th percentile in one cohort of pregnant patients were correlated with a higher likelihood of gestational hypertension, indicating that an excess of aldosterone may also impact gestational outcomes (140). Angiotensin II type 1 receptor agonistic autoantibodies (AT1-AAs) are upregulated in preeclampsia; they bind to and activate AT1 receptors independently of circulating Ang II (141). Sustained receptor activation promotes vasoconstriction, endothelial dysfunction, and hypertension despite relatively low circulating concentrations of renin, Ang II, and aldosterone. Elevated blood pressure and persistent tissue-level AT1 receptor stimulation subsequently suppress renal renin release through negative feedback mechanisms, resulting in downregulation of circulating RAS factors (142). The threshold of RAS factors necessary for a normal pregnancy and the detection of imbalance is under study, and it has yet to be determined whether RAS is a cause or consequence of dysfunctional pregnancy. Lower aldosterone levels may result in a lack of plasma volume expansion and increased stress on the kidneys, whereas a failure to respond to increased estrogen and progesterone may result in fewer podocytes, causing immediate glomerular dysfunction, including glomerular endotheliosis, and elevating long-term risk of maternal chronic kidney disease and fetal renal outcomes (143, 144). Juxtaglomerular cells near the glomerulus help regulate AngII levels: Piezo1 mechanosensors expressed by these cells increase local renal renin production, which in turn increases AngII levels. Piezo1 is also upregulated by inflammatory cytokines, including interleukin (IL)-6, TNFα, and INFγ, which are all elevated in circulation during preeclampsia (145). Elevated tubular epithelial injury markers, including kidney injury molecule-1 (KIM-1) and cystatin C, are also present at higher concentrations in the blood and urine of patients with preeclampsia, likely correlating with ultrastructural damage to the proximal tubule. The gaps left in the proximal tubule by missing and damaged cells result in failed filtration of solutes and proteins, causing them to appear in higher amounts in the urine (146, 147). SGLTs, megalin, cubilin, and other transporters in the proximal tubule are necessary for endocytosis of proteins, glucose, and other solutes, maintaining homeostasis, disruption of which may prevent the normal progression of pregnancy (148–150).

In pregnancy, these diseases further impact the ability of the kidney to respond to the increasing demands of the fetus. They may result in further complicated postpartum disease without adequate management and treatment before, during, and after pregnancy, including monitoring, antihypertensive treatments, and dialysis.

### Maternal Kidney and Placental Interactions

Maternal physiology alone cannot determine the success of pregnancy: fetal response and placental support are also critical to gestational outcomes. The maternal kidneys and placenta have a complex, bidirectional communicative relationship necessary for maintaining a normal physiological milieu for the fetus (151). Forming from the trophoblast of the early embryo after uterine implantation and fully formed by gestational week 20 in humans, the placenta serves as a physical and molecular barrier, attachment point, and exchange organ between the maternal and fetal systems and regulates its claim on maternal supply based on fetal demand (152). The placenta itself uses ∼45% of the oxygen and 30% of the glucose transferred to the uteroplacental interface, demonstrating the high metabolic need to sustain its activities in support of fetal growth (153). Placentation triggers several physiological changes, including adaptation within the maternal kidney. A maternal kidney that fails to respond to the demands of the placenta may adapt poorly, resulting in gestational disease and pregnancy complications.

The placenta secretes several hormones that act on the maternal kidney during pregnancy, including relaxin, estrogen, and progesterone, which alter renal anatomy and functionality as described in previous sections (154–156). As maternal plasma volume increases, the placenta also participates in regulating the renin-angiotensin system by upregulating vasodilation to compensate for increased volume and vascular load (103). The placenta itself has its own independently functioning RAS, which is primarily active in early pregnancy to induce vascularization and gradual trophoblast invasion of the maternal decidua (157, 158). It may also affect circulating levels of AngII by estrogen stimulation of liver angiotensinogen production or by releasing it directly into the circulation itself (36, 159, 160). Elevated maternal aldosterone may be driven by placental estrogen release and thereby affect maternal renal function (161, 162). Slightly elevated AngII in pregnancy does have a purpose: maintaining some vascular tone during pregnancy, especially in the localized uteroplacental region, is critical for the angiogenic remodeling and pruning of blood vessels necessary to maintain maternal-fetal exchange of oxygen and nutrients in human and animal models (163–165). However, excess AngII in the circulation drives global vasoconstriction and maternal renal impairment.

Similarly, the placenta also regulates other angiogenic and antiangiogenic factors that may be released into the circulatory system and impact renal and cardiovascular function. Vascular endothelial growth factor (VEGF) and placental growth factor (PlGF) are necessary for adequate placental development and integration with the maternal decidua (128–131), providing secure attachment and ample exchange with maternal blood. However, increased secretion of soluble fms-like tyrosine kinase 1 (sFlt1) leads to endothelial dysfunction by decoy binding VEGF and PlGF (166), preventing them from reaching their intended receptor targets for placental vascular adaptation (167). Furthermore, sFlt1, specifically elevated in preeclampsia, prevents VEGF binding to maternal renal podocytes and causes reduced GFR, glomerular endotheliosis, and tubular nephropathy (168, 169).

Failed immune response in pregnancy can also lead to gestational complications. The kidney is increasingly recognized to have a rich resident immune system, consisting of macrophages, dendritic cells, T cells, B cells, natural killer (NK) cells, and other lymphocytes (170, 171). Intrarenal immune cells play an important role and homeostasis (172, 173) and can shape physiologic changes to systemic insults such as infection (174) or ischemia (175, 176). The effects of pregnancy on intrarenal immune cells have not yet been investigated, although systemic immune modulations in response to pregnancy are significant (177–179).

Uterine NK cells are critical for implantation (180), and whether NK dysfunction contributes to recurrent miscarriage remains controversial (181, 182). Uterine NK cells show decreased cytotoxicity and immunomodulatory capacity and increased pro-angiogenic functions compared to circulating NK cells (183). Given that the kidney contains a robust population of tissue resident NK cells (184), investigations into pregnancy-induced changes to kidney NK function, and their implications on disease processes (such as preeclampsia or susceptibility to kidney disease later in life) present an area of future investigation. In addition, the placenta secretes a number of cytokines and other factors to recruit cells and macrophages to protect the developing fetus. The maternal immune system becomes more tolerant in a normal pregnancy of the fetus in part because of the immunomodulatory signals released by the placenta to shift the response of maternal T, B, and dendritic cells (185, 186). Maternal systemic inflammatory response is suppressed by placental extravillous trophoblasts which shift the balance of maternal proinflammatory T helper (Th)1 and Cluster of Differentiation (CD)8+ positive cells involved in identifying and destroying foreign bodies to a higher ratio of Th2 and IL-4 anti-inflammatory cytokines (187). Progesterone produced by the corpus luteum in early pregnancy and later by the placenta can further impact the activity of maternal renal NK cells and macrophages (188).

In addition to secreting hormones and regulating the immune system, the placenta also releases extracellular vesicles into circulation that may interact with the maternal kidney. Exosomes are 30-150nm diameter membrane-bound vesicles released by the placenta into the maternal blood containing bioactive molecules that interact with the maternal organs, particularly the kidney (189–191). They are a subset of extracellular vesicles released by placental exocytosis as soon as 6 wk of gestation; they carry miRNA, protein, and other signaling molecules to regulate maternal response to pregnancy (192, 193). Some speculate this is to help regulate the maternal immune system and prevent it from attacking fetal and placental tissues (194–197). However, exosomes may be targeted to discrete maternal organs including the kidney for selective uptake (198). One of the primary ways exosomes regulate maternal renal function is by immune modulation. Exosomes derived from the placental mesenchymal stromal cells (PMSCs) can affect tubulointerstitial fibrosis by increasing Treg differentiation (199). However, exosomes released from placentas in certain pregnancy complications, including preeclampsia, may cause increased proteinuria by affecting glomerular endotheliosis (200). Studies are currently being done to understand the cargo of these exosomes and what targets they have in the maternal kidney (198). These findings underscore the importance of the placenta as an active regulator of pregnancy and maternal renal function, not merely a passive conduit.

## UNRESOLVED QUESTIONS

Maternal renal adaptations in pregnancy and the interconnected response of the maternal kidney and placenta remain incompletely understood. Some of these gaps are illustrated in Fig. 3. In particular, the interactions between the maternal and placental systems, and the necessary checks and balances that match fetoplacental demand to maternal supply, are the subject of current studies aimed at understanding the precise mechanisms and signals mediating their crosstalk. Specific hormones, peptides, and signaling molecules responsible for these adaptations are being assessed in circulation and in the context of extracellular vesicles and exosomes released by the placenta and the maternal kidney (193, 198, 201). The mechanism by which these signals are received is also not fully known. Endocytosis in the kidney and placenta is altered when there are fluctuations in hormones like estrogen or insulin, similar to those observed in gestation (202–205). Specific changes in the activities of endocytic receptors such as megalin and cubilin in the proximal tubule would help us understand the extent of change mediated by signaling mechanisms.

**Figure 3.**
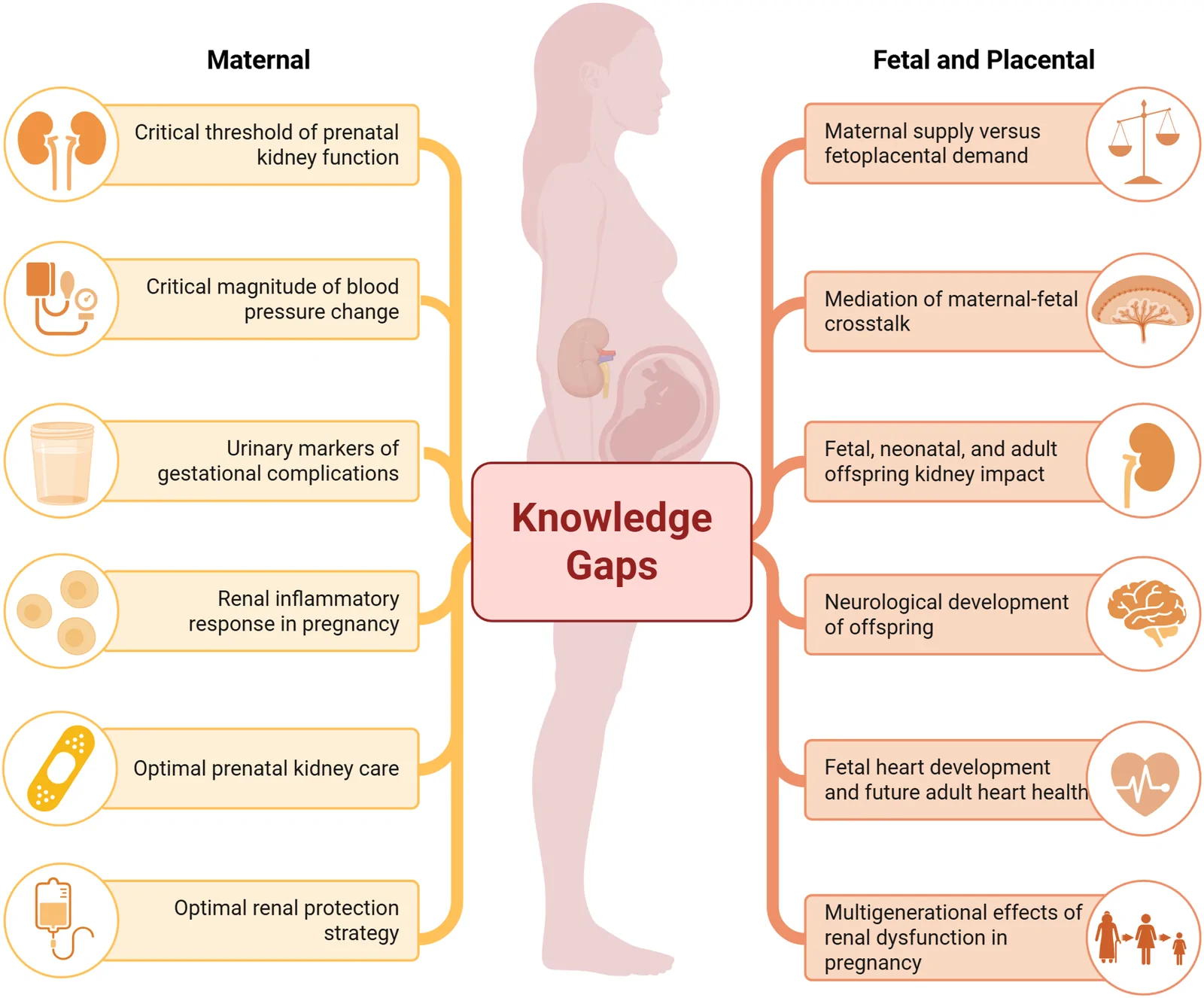
Knowledge gaps for future investigations into maternal and fetoplacental effects of renal dysfunction during pregnancy. Figure created with a licensed version of BioRender.com.

Pregnancy-associated acute kidney injury (AKI) is a significant global health issue that increases maternal and fetal morbidity and mortality, with incidence influenced by socio-economic and healthcare factors. Although it occurs in 1% of pregnancies globally, this rate is higher in low- and middle-income countries; an additional risk factor is pre-existing maternal cardiovascular and metabolic disease (206, 207). Previous maternal history of AKI has been linked to later renal and cardiovascular disease, including pregnancy complications (11, 12), but AKI that occurs in pregnancy has been separately associated with maternal chronic kidney disease, maternal death, neonatal stillbirth and offspring cardiovascular disease (206). Detection in pregnancy is difficult: increased cardiac output, plasma flow, and GFR mask volume depletion and oliguria until maternal and placental dysfunction become evident in late pregnancy (207). New technologies are being developed to assess early changes in maternal renal function during pregnancy and thereby detect pregnancy complications sooner, such as more sensitive methods for clinical reference of GFR, including measurement of urinary cystatin C and subcutaneous analysis of filtration, or the identification of specific serum markers and relevant levels for pregnancy complications like hyaluronan or KIM-1, which may improve our ability to diagnose gestational and postpartum disease (147, 208–211). Omics-based approaches, advanced imaging modalities, and machine learning-driven predictive models are being investigated for their ability to detect early changes in maternal renal function and identify pregnancy complications before clinical disease becomes apparent. Developing improved assays for early detection and therapies for all diseases affecting maternal and fetoplacental functions, including pregnancy-associated AKI, is critical for improving future maternal health outcomes.

From a detection standpoint, one area requiring improvement is determining the critical threshold of maternal kidney function and the magnitude of blood pressure change at which a diagnosis of pregnancy complications is made. With maternal risk factors for pregnancy complications including prenatal obesity, diabetes, and cardiovascular disease that affect baseline pre-pregnancy blood pressure sharply on the rise, deciding the cutoff value for diagnosing preeclampsia and other gestational diseases is becoming more complicated (58, 212–214). How these risks then affect postnatal maternal and offspring disease is also of concern, as increased risk of renal, cardiovascular, and neurological disease is evident years after pregnancy complications (12, 215, 216). Early detection and treatment of maternal renal complications pre-pregnancy or during gestation will improve later-life health.

## CONCLUSIONS

The maternal kidney’s adaptation to pregnancy is a multifaceted process, informed by maternal requirements, fetal demands, and the critical role of placental signaling. Although much has been learned, ongoing research aims to unravel remaining mysteries, enhancing care before, during, and after pregnancy. Considering pregnancy from maternal or fetoplacental perspectives, depending on the lens applied, enriches our understanding of its complexities and guides novel research and clinical approaches.
